# Living with acuteness in chronic illness: The temporal underpinnings of endometriosis

**DOI:** 10.1111/maq.12909

**Published:** 2024-12-28

**Authors:** Venla Oikkonen, Elina Helosvuori

**Affiliations:** ^1^ Tampere Centre for Science, Technology and Innovation Studies Tampere University Tampere Finland; ^2^ Helsinki Collegium for Advanced Studies University of Helsinki Helsinki Finland

**Keywords:** chronic illness, complications, endometriosis, risk, temporality

## Abstract

This article explores how acuteness is experienced by people with endometriosis in Finland. Drawing on in‐depth interviews as well as anonymous written endometriosis stories, we trace instances when the sense of chronicity and cyclicality of endometriosis is disrupted by a possibility of risk to life. These instances include when endometriosis tissue grows in unanticipated and aggressive ways, when medical interventions lead to unexpected complications or medications raise concerns about a gradually developing risk, and when endometriosis diagnosis becomes a catch‐all category that could mask the onset of a life‐threatening condition. Our analysis of illness experiences suggests that, while risk to life is an unlikely outcome in chronic conditions such as endometriosis, concerns about risk shape how the chronicity and cyclicality of endometriosis are felt and managed in everyday life.

## INTRODUCTION

Chronic illness has emerged as a phenomenon with increasing societal and scholarly significance in recent years. Medical anthropologists have examined the ways in which chronic illnesses are reshaping how health, illness and quality of life are assessed, managed, and lived with (e.g., Greco & Graber, 2022; Svensson et al., 2020; Wahlberg, 2018). A central theme in this literature is temporality. Several scholars have questioned the boundary between acute and chronic conditions on which biomedicine operates, showing that chronic illnesses often involve moments of acuteness (see Greco & Graber, 2022; Manderson & Smith‐Morris, 2010; Whyte, 2012). For example, long‐term trajectories of chronic illness may be interrupted by unexpected, rapidly evolving health concerns.

Endometriosis is a common chronic gynecological condition in which tissue similar to the uterine lining grows outside the uterus causing pain, gastrointestinal symptoms and fertility problems. While biomedical literature acknowledges that endometriosis symptoms can affect a person's life in severe and debilitating ways, endometriosis is nevertheless understood to be a nonlife‐threatening disease and is therefore described as a “benign gynecological condition” (Pavone & Lyttle, 2015; Pynnä et al., 2021; Westwood et al., 2023) or a “benign uterine disorder” (Vannuccini et al., 2022). These framings arise from comparisons to acute, life‐threatening conditions that require immediate medical attention.

Yet, we suggest, many people's experiences of living with endometriosis are structured by the possibility of risk to life. This possibility is distant yet tangible. It haunts the experiences of living with endometriosis, appearing and reappearing in often unexpected moments. Such moments interrupt the sense of chronic temporality as well as the cyclicality of menstruation‐related endometriosis symptoms. While biomedical literature on endometriosis foregrounds its nonlife‐threatening nature, there is a specific strand within this literature that examines, through case reports or literature reviews, the circumstances in which endometriosis may become life‐threatening. Endometriosis tissue may grow in places where it results, for example, in a ruptured intestine or a ruptured endometrioma (ovarian cyst), conditions that carry a risk of serious bacterial infection and sepsis (Foote et al., 2023; Goriel et al., 2023; Petruškevičiūtė & Bužinskienė, 2021). Endometriosis growth can also block an intestine or urinary tract, or result in a twisted ovary, which may cause tissue damage by stopping blood flow to the affected organs (Coutureau et al., 2023). While these complications are rare, awareness about their existence structures illness experiences.

Risk to life is also present in less tangible ways. Endometriosis has been compared to cancer in terms of how it spreads, affecting multiple organs especially in the abdominal cavity. Endometriosis “has features similar to that of malignant tumors including the ability to develop distant foci” (Pavone & Lyttle, 2015, 663), while it differs from cancer in that endometriosis cells are “morphologically normal, but misplaced” (Chui et al., 2017, 78263). At the same time, the connection between endometriosis and cancer extends beyond a possible likeness in how they spread. Biomedical studies have suggested that endometriosis may increase the risk of some malignant tumors such as ovarian cancer (Chui et al., 2017; Pavone & Lyttle, 2015). Thus, the association between endometriosis and cancer structures illness experiences also as a question of illness trajectories that could potentially unfold from the present.

For most people with endometriosis, these perceived risks to life are felt rather than realized as actual medical emergencies. Yet, we maintain, the sense of potential even if unlikely danger shapes how endometriosis as a chronic illness is experienced by those with debilitating or unexpected symptoms. That the boundary between endometriosis pain and “normal” period pain has been contested in society and in clinical practice (Hudson, 2022; Jones, 2016; Seear, 2014) contributes to the experience of danger. Uncertainty about what constitute normal period‐related symptoms can make it challenging for someone with endometriosis to evaluate which of their evolving symptoms need medical attention (see Rogers, 2022 for slippery disease categories). This is why endometriosis provides a particularly illuminating viewpoint into the dynamic of acute and chronic conditions in long‐term illness.

Drawing on interviews with people with endometriosis and anonymous written endometriosis stories collected in Finland, we ask how the idea of risk to life shapes the temporality of endometriosis as a lived, embodied illness. We situate the data in relation to medical anthropological and medical sociological literature on the temporality of chronic illness and the temporality of period‐related pain, including endometriosis. We show that experiences of risk to life in endometriosis result in a temporal reorientation that complicates both the experienced chronicity and cyclicality of chronic gynecological illness. The article contributes to the medical anthropological scholarship on temporality by elucidating how the tensions between chronicity, cyclicality, and acuteness in endometriosis structure experiences of illness and its treatment.

In what follows, we first introduce our theoretical framework, focusing on temporality in chronic illness. The three analysis sections that follow are organized around three themes we identified in the data: (1) endometriosis as a threat to life, (2) the treatment of endometriosis as a threat to life, and (3) fear that endometriosis diagnosis may lead to misdiagnosis of other, life‐threatening conditions. Throughout the sections, we focus on moments when risk to life pushes against the sense of chronicity and cyclicality of chronic gynecological illness, as well as trace the ways in which our interlocutors manage and anticipate these situations.

## CHRONICITY, CYCLICALITY, AND ENDOMETRIOSIS

According to Jowsey (2016), scholarship exploring the interrelationship between temporality and chronicity focuses on four temporal issues: calendar and clocked time, biographical time, past–present–future time, and inner time and rhythms. While the first three address social temporal structures, the fourth one is an issue of the temporality of the body. Our research is aligned with the last category: we explore moments of threat to life through the prism of embodied experience and inner rhythms of the body.

Our approach resonates with medical anthropological studies that highlight how the dichotomy between acute and chronic illnesses fails to acknowledge lived experience of time in chronic illness (see Greco & Graber, 2022; Manderson & Smith‐Morris, 2010; Whyte, 2012). This literature shows that people's orientation to time in chronic illness is situated and changing. For example, chronic illnesses may include a sense of acuteness during intense and possibly invasive clinical examinations and tests (Jowsey, 2016, 1098). The appearance of comorbidities may also engender a sense of “recursive cascades” that complicates steady chronicity (Manderson & Warren, 2016). As we will show throughout this article, the experience that life‐threatening events are possible in endometriosis involves a reorientation of time that interrupts the sense of an unfolding chronic illness trajectory. Our analysis of how these moments of reorientation are felt and managed contributes to the critiques that maintain that the relationship between acute and chronic conditions is unfixed and evolving (Greco & Graber, 2022; Manderson & Smith‐Morris, 2010; Whyte, 2012).

The specific nature of embodied orientation and temporality in “chronic living” (see Wahlberg, 2018) has been explored from different perspectives. Poleykett (2023) examines how the onset of chronic conditions such as diabetes, hypertension and heart disease reshapes perceptions of time, embodiment, ageing and the life course in the Senegalese city of Dakar. Poleykett (2023, 77) argues that new forms of embodied temporality produced and engaged by ailing and ageing people emerge in the wake of “new diseases”: chronic symptoms may appear out of time, at unexpected junctures in the life course. Furthermore, chronic conditions are understood to speed up or accelerate the rhythm of the body (Poleykett, 2023). Other studies have shown how chronic, incurable pain in the body shapes identity (see Gotlib, 2013), and how chronic conditions such as diabetes alter the horizon regarding what can be expected from the body that needs extensive self‐management and medical control (Morris, 2008).

Some medical anthropologists have argued that developments in biomedicine and biotechnology render conditions as chronic in new ways (see Greco & Graber, 2022; Wahlberg, 2018). Exploring the role of technological devices, Barlocco (2022) suggests that the use of the implantable cardioverter defibrillator (ICD) in cardiac diseases to prevent sudden death contributes to the development of a particular type of chronicity. Embedded into patients’ bodies, ICDs allow patients to form an identity in relation to not only the disease but also the technology. Barlocco argues that discussion focusing on risk, quality of life and patient choice “leaves the patients lacking resources to make sense of their experience of chronicity in a body that at a certain point becomes diseased and then inhabited by a machine” (Barlocco, 2022, 62). We draw on this strand of research in that we analyze the role of diagnostic tools and imaging technologies in how people with endometriosis manage uncertainties about the seriousness of symptoms and enact boundaries between harmless and life‐threatening embodied processes.

Endometriosis has its own specific temporal underpinnings. In endometriosis, chronic time intersects with cyclicality as the worst symptoms typically occur during menstruation. Scholars have noted that this temporal association between pain and menstruation has led to the misdiagnosis of endometriosis as menstrual pain (Bullo, 2018; Griffith, 2020; Hudson, 2022; Jones, 2016; Seear, 2014). The focus on menstrual cyclicality has also sidelined the chronicity of endometriosis as an incurable, often progressing illness (Hallström, 2024; Jones, 2016). Feminist scholars have challenged this framing, highlighting that endometriosis is, first and foremost, a gendered chronic illness characterized by a specific form of *cyclic chronicity*. That is, the cyclicality of endometriosis takes place within a temporal framework of chronicity. Przybylo and Fahs (2018) argue that menstruation‐related pain, such as endometriosis pain, “demands new theorizations of pain temporalities” that account for both the cyclicality and chronicity of pain (Przybylo & Fahs, 2018, 208). They note that cyclic chronicity renders menstruation‐related pain “familiar and unfamiliar, expected and unexpected, shifting our sense of time” (Przybylo & Fahs, 2018, 219). Jones (2016) argues that chronicity is so central to endometriosis pain that endometriosis should be seen as a question of gendered disability. Like Przybylo and Fahs, Jones focuses on the specific qualities of endometriosis pain that break the sense of steady chronicity and safe cyclicality.

Hallström (2024) proposes the concept of *endo time* to capture the multiple temporal configurations that characterize the experiences of living with endometriosis. Building on phenomenological approaches to temporality and embodiment, Hallström distinguishes three temporalities. *Waiting time* refers to the ways in which the waiting for diagnosis and medical recognition – which may take years, even decades – puts everything else in life on hold, suspending the sense of futurity (Hallström, 2024). *Cyclical or chronic time* refers to two patterns of symptoms and their societal recognition (Hallström, 2024). Cyclically occurring symptoms associated with menstruation are dismissed as normal period pain that one should learn to live with, whereas debilitating symptoms that appear outside the periods are perceived as a personal failure (Hallström, 2024). Both cyclical and chronic time encourages a person with endometriosis to conceal the extent of their symptoms when faced with social expectations about productivity. Finally, *sedimented time* refers to the way in which the embodied histories of endometriosis, such as previous experiences of uncontrollable bleeding or inadequate treatment of endometriosis pain, shape how endometriosis is lived with in the present, resulting in a tension between embodied experience rooted in the past and future‐oriented societal rhythms (Hallström, 2024). Time is also sedimented in the sense that the passage of time shapes the body materially, for example, when endometriosis lesions spread, or pain becomes chronic, because of diagnostic delay (Hallström, 2024).

While building on this body of literature, we focus on moments of rupture engendered by perceived threat to life. We explore how the possibility of risk to life interrupts both the chronicity and cyclicality of endometriosis. The analysis examines how these moments of rupture arise in relation to sedimented past experiences and the materiality of the body (Hallström, 2024) and how they turn the attention to the intensity of the present. Furthermore, we trace how our interlocutors respond to these temporal ruptures by enacting boundaries between life‐threatening and harmless symptoms.

## DATA AND METHODS

This article presents findings from a social science research project on three gendered chronic illnesses in Finland. The data collected within the endometriosis subproject includes interviews with clinicians, patient activists, biomedical researchers and persons living with endometriosis, ethnographic observation at an endometriosis clinic, biomedical literature and treatment guidelines, patient organization support materials, news stories on endometriosis, as well as anonymous endometriosis stories. In this article, we draw on 27 interviews with people with endometriosis and 43 anonymous written stories of living with endometriosis. The data was collected by both authors in Finland in 2021–2023. The semi‐structured interviews covered, among other themes, the path toward diagnosis, decisions about treatment options, and sources of information on endometriosis. We received written informed consent from all interviewees. The written stories were collected with an anonymous online form, posted on the project website, in which we asked people with endometriosis to write freely about any aspect of their illness while we also listed the same themes that were covered in the interview data as possible topics. Both the interview participants and the anonymous writers were able to cancel their participation at any time. We have removed all identifying details to guarantee anonymity. All names are pseudonyms created solely for this article.

The call for interview participants and the call for written stories were posted on our project website and Twitter account and shared by a national patient organization through their channels. Using online platforms allowed us to engage with people living in different parts of the country. Our interviewees are fluent speakers of Finnish or Swedish. The illness stories were written in Finnish. It is important to note that a call for endometriosis experiences is likely to result in a higher‐than‐average number of accounts of complicated cases of endometriosis. While there are many serious illness histories in our data, the data also includes milder cases of endometriosis.

Risk to life came up in several interviews and written stories. It is present in our data both as a physiological process and as a metaphor, such as “death” of social life, career ambitions or relationships, or in concerns about the effects of endometriosis on fertility. In this article, we focus on physiological processes that threaten the life of the person with endometriosis. We analyze instances in which dying as a physiological event is referred to. Some of these references are direct and discuss past life‐threatening incidents or a future possibility of a life‐threatening complication. Other references are indirect: risk to life may be present in alternative scenarios invoked by our interlocutors—what could happen or could have happened – or in affective intensities such as concerns attached to medical procedures or the progression of endometriosis. Nevertheless, even when present as an idea, risk to life still refers to a physiological process instead of being a metaphor for the narrowing of biographical prospects.

The analysis involved several rounds of close reading by both authors. After considering which mentions of danger filled the selection criteria described above, we re‐read these mentions multiple times to detect intersecting themes. We identified three aspects of risk to life in the data: endometriosis threatening life, endometriosis treatment threatening life, and endometriosis diagnosis leading to misdiagnosis of other, life‐threatening conditions. The three findings sections that follow are organized according to these themes. Within each section, we view the theme in terms of how the risk to life ruptures the sense of steady or progressing chronicity or expectations about cyclical patterns of symptoms.

## WHEN ENDOMETRIOSIS THREATENS LIFE

As mentioned in the introduction, endometriosis is sometimes compared to cancer in terms of how it spreads, while a distinction is made between the two in terms of endometriosis being primarily chronic and cancer being life threatening (Chui et al., 2017; Pavone & Lyttle, 2015). Comparisons to cancer are also present in our interview and story data; however, the boundary between endometriosis and cancer appears situated and evolving rather than stable in embodied experiences of living with endometriosis. The juxtaposition of endometriosis and cancer provides a sense of relief in some situations, yet it also raises concerns as it highlights the potentially expansive and unpredictable growth of endometriosis. For example, Tilda, in her mid‐30s at the time of the interview, invokes breast cancer as the context in which she makes sense of her endometriosis: “There is breast cancer in my family, and I see endometriosis as a similar rhizome‐like growth that spreads in the body and is uncontrollable and scary. Although it's not life‐threatening, I understand that there is a similarity in how it spreads.” Positioning her endometriosis within the temporal context of past (and potential future) cases of breast cancer in the family, Tilda emphasizes the benign nature of endometriosis yet sees the unpredictability of endometriosis as “scary.”

In another interview, Kaarina, who is in her late forties, uses similar imagery of rhizome‐like expansion to describe the effects of endometriosis inside her body. During an operation to remove her uterus and ovaries, surgeons had discovered a series of pathological changes in the pelvic area, including fibroids and wide‐spread endometriosis lesions:

My uterus was about three times the weight of a normal uterus. It was clearly enlarged. And the bladder was shaped like an eight, endometriosis tissue tied the uterus, the bladder and an old C‐section scar together. There was adhesion of tissue in all three, and it took the surgeons a long time to separate them. They couldn't remove the uterus until at the end of the operation because they had to first detach the bladder from the uterus. Now the bladder is supposed to be back where it should be. And then the right ovary, there were never any symptoms on the right side, but endometriosis tissue had attached the ovary to the pelvis.

Kaarina describes endometriosis as a quietly progressing condition that may deceive both the patient and the clinicians. Like cancer, its effects may not be fully felt by the patient until the illness reaches a critical point. Neither is it always captured by imaging technologies or other tests, positing surgery as a moment when the extent of the tissue damage caused by endometriosis is revealed.

While a distinction is typically made between life‐threatening cancer and nonlife‐threatening endometriosis, the latter can, in some circumstances, become life‐threatening. In Kaarina's case, the surgeons doing the operation also discovered an acute condition: endometriosis growth had partially blocked an intestine. Preoperative imaging tests had not revealed the seriousness of the situation. Instead, Kaarina's sensations of pain at the site of the blockage had been the only indication that the chronic, gradual progression of endometriosis had resulted in an acute situation that required medical intervention. The operation included gastrointestinal surgery, which aimed to halt the expansion of the endometriosis tissue. The aggressive growth of endometriosis tissue contradicts the expected chronic temporality of a body affected by a nonlife‐threatening long‐term illness. Crucially, the expansion of the endometriosis tissue remains invisible to the eye and thus comes as a surprise during surgery.

Seela, in her late forties, also challenges the assumption that endometriosis is always chronic and only cancer is life‐threatening. Oral contraceptives are among the most widely used treatments for endometriosis as they help control the body's estrogen level, which is linked to endometriosis symptoms. In other words, hormones are used to minimize the cyclicality of the menstruating body. In an interview, Seela describes how half a year after stopping the pill for health reasons, she experienced quickly worsening symptoms. She was treated with several courses of antibiotics for what was assumed to be a urinary tract infection, but her health got worse. Eventually, after a number of clinical visits, tests and medical procedures, it was discovered that endometriosis had created a hole in her intestines: “Everything went to a wrong place and had caused the infection. So it wasn't a urinary tract infection. When they began to suspect that there was a hole, I had an emergency operation. It had to be fixed of course because if nothing is done, I understand that you will die of infection.” The unpredictability of endometriosis tissue invoked by Kaarina is also present in Seela's case, although it takes a different shape. Instead of blocking an intestine, it makes a hole in healthy tissue, breaking material boundaries necessary for the maintenance of human life. Drawing on her experience, Seela reflects critically on the comparison between endometriosis and cancer: “A doctor told me that it [endometriosis] is like cancer but it doesn't kill, it spreads and causes pain and other things. Although in my case it almost tried to kill me, but thankfully it didn't manage.” The rupturing of the intestine challenges the distinction between acuteness and chronicity. The continuation of a personal chronic illness trajectory is shown to depend on the ability of the healthcare system to recognize and address the acute complication resulting from the unpredictable behaviour of endometriosis tissue.

In the above examples, the potential danger is fully known only in retrospect. The moments of crisis that lead to the discovery of the potentially escalating health issue show how uncertain knowledge can be at the moment of discovery. This uncertainty is visible in a written endometriosis story by Aada, in her early forties, who describes how the boundary between endometriosis and possible cancer becomes blurred in the practices of testing. After her period‐related pain had grown in intensity, Aada decided to see a doctor despite being concerned that her symptoms would be dismissed—a decision that drew on her past experience of what constitutes “normal pain” in her case:

They tested my CRP for infection, and it was over 100 at that time. I was sent immediately to the hospital, and after several tests and an MRI was admitted to the gynecology ward. I was operated during the same visit because the infection was caused by an endometrioma that was 10 centimeters in diameter, had started bleeding and had made the markers for ovarian cancer rise dramatically.

In addition to removing the endometrioma, the surgery involved removal of a section of the intestine and numerous endometriosis lesions as well as blood that had accumulated around the abdominal membrane and the liver. While infection related to endometrioma can be dangerous if not treated, here the threat to life is present also in another way. Aada's endometriosis is not just cancer‐like in its growth pattern but also, in the diagnostic tests conducted at the hospital, indicates a potential alternative diagnosis of ovarian cancer. This results in multiple potential futures opening up, some with chronic and some with life‐threatening implications. Crucially, a technological intervention—testing—is not sufficient to establish a firm boundary between safe and dangerous symptoms. At the same time, the health crisis itself is tied to the cyclicality of menstruation, as Aada's symptoms had worsened during menstruation and the associated changes in estrogen levels. Likewise, Aada's postoperative treatment aims at removing periods with a hormonal product. The underlying cyclicality of highly concerning endometriosis symptoms is repeated in other accounts: for example, Seela also suffered from a collapsed lung that appeared only during menstruation and was presumably caused by endometriosis tissue around the lung.

Our data suggests that experiences like these can have lasting effects on personal perception of risk and of the temporal progression of one's illness. Experiences of potentially life‐threatening complications from endometriosis may shape how people react preemptively to potential future crises. For example, Kaarina had experienced uterine hemorrhage and had been taken to the hospital in an ambulance and treated with coagulants. The experience made her decide to have a full radical surgery (removal of the uterus and both ovaries) to prevent any future occurrence:

The doctor said that they recommend radical surgery, that everything is removed, but that I can decide […] whether I want to keep the right ovary. The good thing would be that the ovary might keep hormone production going. Until the uterine hemorrhage, I considered whether to remove only what is necessary. After the hemorrhage I told my partner that now it's clear that everything should go. It can't continue like this, that there would be symptoms still after a partial surgery.

The uterine hemorrhage challenges the assumption that endometriosis symptoms are not life‐threatening – and thereby the presumed chronic temporality of endometriosis as an illness. It also mobilizes sedimented time (Hallström, 2024) in the form of worried anticipation and precaution arising from embodied experience of ill health. Sedimented time reorients personal hopes about future illness trajectories and is reflected in the decision to undergo full radical surgery. The health crisis also makes visible the underlying cyclical patterns of endometriosis: while a partial operation could keep the body tied to the cyclical patterns of hormone production, radical surgery is posited as opening a trajectory that is free from potentially recurring symptoms. Radical surgery, then, is understood to enact and stabilize a boundary between safe and risky biological processes.

## WHEN ENDOMETRIOSIS TREATMENT THREATENS LIFE

In our data, two treatment‐related risks appear: unexpected complications of surgery and cancer‐risk associated with some hormonal pharmaceuticals.

Mentions of risks from surgery are relatively common in our data. For example, an endometriosis story written by Ulla, in her mid‐forties, mentions surgery‐related risks as part of a long list of problems with endometriosis treatment: “Numerous hormone treatments, a shocking amount of money spent on this illness, 16 operations (death wasn't far during a couple of them), uterus and ovaries removed, prolapse operation resulting in nerve damage.” Another endometriosis story by Leena, in her early 30s, describes a complication after an operation to remove an endometrioma:

There was bleeding into the stomach cavity and it led to clotting, which caused an infection. So I was in hospital twice and had a long course of treatment with a strong antibiotic. Altogether it took about a month to recover from the operation. During the operation they found plenty of endometriosis in the wall between the vagina and the rectum, which couldn't be removed without a gastrointestinal surgeon because the operation was so demanding.

Here, as in several other interviews and stories, surgical operations are portrayed as unpredictable. In addition to revealing the hidden doings of endometriosis tissue, as seen in the previous section, the course of the operation may involve unforeseen medical turns. In Leena's case, the technical difficulty of the operation and the need for a gastrointestinal surgeon became apparent only during surgery, a pattern mentioned in several interviews as well.

In the cases described in the previous section, endometriosis itself unsettles the assumed divide between acute and chronic conditions by growing in the wrong place (such as intestines) or at the wrong speed (too aggressively), while medical intervention, especially surgery, provides relief. In the examples in this section, however, the event of surgery posits a risk that may trigger a chain of cascading events. Sanna, in her early forties, describes in an interview the acute illness trajectory that started with what was expected to be a removal of a fallopian tube damaged by endometriosis:

If you read the medical case summary, during the operation the situation turned out to be much worse than expected. They had had to call additional medical staff to the operating room, and, in the end, they removed both fallopian tubes and the operation lasted over four hours. […] After the surgery, I was home for maybe five hours. I can't remember anything other than that I had a high fever already then, and I was lucky to have a friend who had brought me home from the hospital. I was taken back to the hospital in an ambulance, I was on intravenous therapy for over a week, and my parents were told that the situation looks bad, that the antibiotics are not working. I had been sent home because no one had read the medical note or received the information that it had been a massive operation, not an outpatient procedure.

A risk to life unfolds here in a series of events that begin with the surgery and involves erroneous assessment of the medical situation. When antibiotics do not work as they should, death appears as a previously inconceivable but suddenly possible outcome.

In light of experiences like these, it is no wonder that several interlocutors attach a sense of danger to endometriosis surgery. Paula, now in her late 30s, recalls in an interview how she experienced a sense of foreboding before undergoing surgery in her early 20s: “I went to the surgery and had a feeling that everything was not okay. At the last minute, I was told that [trusted endometriosis specialist] will not be doing the surgery after all, instead I'll be operated by a cancer doctor and a gastrointestinal surgeon.” She continues: “It was a very difficult operation, it lasted eight hours” and had to be discontinued because “my heart wouldn't have been able to tolerate general anesthesia any longer.” When she woke up after surgery, she found out that she had been given a stoma, an opening in the abdomen for removal of feces. She notes that this relatively early event in her long history of living with an aggressive case of endometriosis has influenced her concerns about medical interventions, especially surgeries. As in the example in the previous section, the sedimented time of the embodied experiences of illness structure her wishes about future treatment as well as her encounters with clinicians also outside endometriosis care, for example, during her subsequent pregnancies.

A distinctly different temporal configuration emerges from concerns about the long‐term effects of hormonal endometriosis medications expressed by some interlocutors. The long‐term medications used in endometriosis treatment range from hormonal products used to halt menstruation and period‐related symptoms, to hormone therapy used after removal of ovaries to provide a controlled level of estrogen. Hormonal contraceptives and especially menopausal hormone therapy are conceptualized by some interlocutors within a framework of cancer risk as well as chemical overload. These risks may, or may not, unfold over years or decades to come. Irina, in her late 30s, describes in an interview the reasoning behind her decision after her surgery to opt for a transdermal hormone patch instead of an oral hormone product:

I don't want an oral hormone product that might cause harm to the liver. I was also worried about breast cancer risk because hormone replacement therapy increases that risk. So we chose the patch, and in addition to that I got [name of an estrogen gel], a gel that I can use if I need it.

Cancer risk is a long‐term concern that stands in stark contrast to the sense of the “now” associated with unexpected surgery complications. Likewise, concern about damage to the liver from extensive use of pharmaceuticals – a concern shared by other interlocutors – posits chemical exposure as a gradual process that may impact future health.

Concerns about effects of medications on future health are not unique to endometriosis, for example, the link between menopausal hormone therapy and cancer is a long‐standing worry among women (Fishman et al., 2015; Lupton, 1996). However, when used in the treatment of endometriosis, menopausal hormone therapy plays a specific temporally invested role. It replaces the chronic cyclicality of the preoperative body (a source of endometriosis symptoms) while establishing a sense of a steady postoperative trajectory from a chronically ill past to an endometriosis‐free future. When people with endometriosis negotiate postoperative hormonal care, as in the example above, their treatment options are positioned in relation to these temporally invested concerns about chronic illness trajectories, their cyclical underpinnings, and possible though unlikely life‐threatening heath issues arising from different medications.

## ENDOMETRIOSIS DIAGNOSIS MASKING LIFE‐THREATENING CONDITIONS

In our data, concerns that endometriosis could mask life‐threatening medical issues are discussed especially in relation to ruptured appendix and malignant tumours. Medical emergencies such as ectopic pregnancies also appear as issues against which endometriosis symptoms are contrasted.

Tyyne, in her mid‐forties, explains in an interview that she is concerned that abdominal pain associated with her endometriosis might lead to missing the symptoms of a possible appendicitis. This concern has become part of her work life. She has told her colleagues about her endometriosis and has had pain attacks during office days. She describes how in these situations she may ask her colleagues for help to make sure that the pain really is caused by endometriosis:

It has been more than once or twice that a colleague has come to my office and applied pressure to my stomach so we can find out whether it's endometriosis or appendicitis. You lie on your back and press down on the stomach and let it bounce back. If the bouncing causes acute pain, then it's the appendix, but if it doesn't, then it's endometriosis.

Through this technique, which she learned from a physician, Tyyne has sought to rule out the possibility that pain symptoms are caused by a severe complication requiring immediate medical care. With the help of her colleagues, she decides whether it is safe to medicate the pain instead of rushing to the emergency room.

The alarming intensity of pain attacks is described vividly across the interview and story data, which includes several descriptions of losing—or being very close to losing—consciousness because of pain. Such pain is scary. These incidents may include fear of dying, as in the following description in an interview with Krista, in her mid‐forties, of a visit to a family summer cottage:

And there it happened. I was carrying a black trash bag, then I got this extreme pain attack. I was certain that I would die there. I felt like throwing up. […] My mother drove me to the doctor's. […] My CRP was going up and then they decided that I would go to [name of hospital]. There they weren't sure if it was the appendix because the symptoms were not quite consistent with it.

During episodes like this, risk to life is felt as acutely present and yet open, as it may be unclear whether symptoms are caused by endometriosis or another condition. According to our interlocutors, debilitating pain and uncertainty about its source also strongly affects family members and friends, who have to decide whether to call an ambulance.

Some interlocutors have tried to get pregnant while at the same time managing the evolving symptoms of endometriosis. This made them worry that serious pregnancy‐related conditions could be mistaken for endometriosis. In the following interview excerpt, Sirpa, in her late 20s, describes how symptoms that could indicate ectopic pregnancy were attributed to her endometriosis diagnosis:

It's thought that endometriosis can cause anything and therefore there's no reason for further action. It's because of the illness, and no matter how severe the pain, the symptoms don't need to be treated because it's supposedly known what causes it. It doesn't require further tests. Yet, for instance in my case in the summer [visiting the emergency room because of severe pain], yes, I have endometriosis, but it didn't exclude the possibility that I could have ectopic pregnancy as I'm trying to get pregnant. Endometriosis also increases the likelihood of ectopic pregnancies. Concerns were raised about it when my need for care was evaluated, but the doctor decided that the pain attack was caused by my [endometriosis] diagnosis.

The excerpt shows how concerns about acute symptoms may be raised and yet dismissed in clinical settings because endometriosis is assumed to include a range of unusual symptoms. Yet life‐threatening conditions, such as ectopic pregnancies, may occur despite of having a chronic condition—a chronic condition may even increase the likelihood of an acute condition. As noted earlier, sedimented time emerges from the embodied experiences of living with endometriosis and the illness molding the body (Hallström, 2024). However, sedimented time is not only drawn on but also renegotiated when new kinds of ordeals appear: the possibility of ectopic pregnancy demands patients’ as well as clinicians’ attention and action.

As noted in the first empirical section, the possibility of malignant tissue transformations hiding beneath benign medical findings is something that also the clinicians need to keep an eye on. The next quotation from an interview with Varpu, who is in her forties, describes how sometimes biopsies need to be taken to exclude dangerous mutations:

They took a sample as we had agreed, something related to the uterus, what is it now… Pipelle biopsy, because my tumour markers were high. And then it was decided that the test for tumour markers would be repeated. At that point I began to be a little afraid when they said that I have nothing, yet I had such awful pain and I had fever. I had a long stretch of fever without cold symptoms.

Comforting as it is to find out that rising tumor markers are caused by endometriosis, a single test may not provide an immediate answer but may need to be repeated, as in Varpu's case. While repeated tests may eventually address concerns about hidden life‐threatening conditions, they do not resolve such concerns immediately and may cause worry.

In addition to repeating tests, Varpu also had to wait for the biopsy results. Indeed, the “waiting time” (Hallström, 2024) for test results that could rule out life‐threatening conditions may involve weeks of uncertainty. Irina describes vividly such processes of waiting:

It was maybe October when I had the appointment. I had an MRI scan and an ultrasound exam. During the time I waited, the endometrioma in the left ovary had grown. It had been a couple of centimetres at the beginning of the summer, and it was six, seven or eight centimetres in October. It had grown a lot during that time. For that reason, I was tested for cancer markers to make sure it wasn't something else than endometrioma as it had grown so fast. The markers were slightly raised. But because endometriosis also raises the markers it was assumed that yes, it's endometriosis and not cancer.

Here the passage of months increases rather than settles the uncertainty about whether the endometrioma is caused by endometriosis or whether it is an ovarian tumor. As tests cannot resolve the uncertainty, the clinical understanding of endometriosis as a catch‐all category for symptoms is mobilized to render cancer as unlikely.

The fact that relatively harmless endometriosis lesions may raise the cancer markers used in medical assessment of symptoms causes concerns in patients, as with Irina above. Indeed, one of the characteristics of endometriosis is that its symptoms may resemble those of grave conditions. In addition to raised tumour markers, some of our interlocutors have experienced, for example, episodes of intestinal bleeding that they saw as highly alarming because it could indicate a threat to life from an undiscovered acute illness. Some episodes have led them to seek medical evaluation at the emergency ward. Episodes of internal bleeding together with a low iron level of blood is an issue that among our interlocutors had required intestinal endoscopy to gain certainty that endometriosis was still the reason for the symptoms. Awareness among people with endometriosis that symptoms like internal bleeding or a new type of intense pain could be caused by another condition keep the line between acute and chronic illness open. What may appear as an acute episode—an intensification of the present – within a chronic illness trajectory, could potentially turn out to be part of an entirely different illness with a life‐threatening trajectory.

## CONCLUSION

This article has explored how risk to life emerges as a felt and lived possibility in endometriosis—regardless of the biomedical framing of endometriosis as a “benign” condition. In the interviews and stories we have discussed, the cyclicality of endometriosis disappears or becomes unsteady and unpredictable. Furthermore, the long‐term trajectory of living with chronic illness is renegotiated as risk to life appears on the horizon as a concern. At the same time, acuteness is enacted in relation to cyclical and chronic temporal orientations. For example, cyclicality of pain may indicate that a symptom is menstruation‐related and thus nonlife‐threatening, but cyclicality may also contribute to the expansion of endometriosis tissue at bodily sites where it may eventually cause complications.

Our study confirms that although patients find comfort in knowing that their disease is not fatal as such, the lived temporality of endometriosis as an illness is also shaped by concern about life‐threatening developments. This fear is rooted in medical emergencies, sedimented embodied history of illness and treatment, and episodes that could have had tragic outcomes. Sudden alarming symptoms, the unknown and hidden growth patterns of endometriosis tissue, and the waiting time involved in testing for alternative diagnoses engender worry. Embodied temporalities of living with endometriosis include an interplay of not only chronic and cyclical, but also immediate and acute reorientation toward time. This orientation is rooted in the embodied ordeals that are either caused by endometriosis, its treatment, or symptoms related to illnesses that might be lurking beneath the already diagnosed condition.

Previous medical anthropological and related literature has explored the complexity of time in chronic illness (Barlocco, 2022; Greco & Graber, 2022; Manderson & Smith‐Morris, 2010; Poleykett, 2023; Whyte, 2012) as well as the intertwining of chronicity and cyclicality in period‐related pain (Hallström 2024; Jones 2016; Przybylo & Fahs, 2018). Our analysis contributes to this scholarship by showing how, in endometriosis, the possibility of risk to life engenders a particular sense of acuteness as well as future‐oriented concern that shapes how chronicity and cyclicality are experienced. The analysis sheds new light on the rhythms of the chronically ill body (see Jowsey, 2016) by showing how the material expansion of endometriosis lesions over time and the lingering of past experiences of pain shape how moments of acuteness are experienced at the intersection of everyday and clinical settings.

The analysis has illustrated how the fact that also illnesses understood to be benign may cause life‐threatening complications structures “chronic living” (Wahlberg, 2018). The unpredictable characteristics of endometriosis tissue constitute a temporal orientation that may run against the expected chronic temporality of a body affected by a long‐term illness. The unfolding of endometriosis in the body may materialize as medical emergencies requiring immediate treatment. As sedimented experiences, these acute episodes have a temporal structure that differs from chronic and cyclical orientation toward time.

All in all, our analysis shows how risk to life appears as an unresolved presence that pauses the sense of chronicity and cyclicality through both immediate health emergencies and concerns about long‐term illness trajectories. Focusing on illness experiences makes visible that the felt presence of risk shapes illness temporality also beyond the moments of actual, physical danger through sedimented fears about possible future complications and the need to observe alarming changes in symptoms in the future. Finally, our analysis has touched only briefly on the role of pain in how time and risk are perceived. We propose that the link between pain and risk to life in the temporality of endometriosis merits further analysis. That changes in symptoms often need to be evaluated while in severe pain inevitably complicates the ways in which the possibility of life‐threatening developments is experienced as a temporal orientation.
